# A model for the management of difficult to treat depression

**DOI:** 10.1192/j.eurpsy.2021.136

**Published:** 2021-08-13

**Authors:** H. Mcallister-Williams

**Affiliations:** Mental Health, Dementia And Neurodegenerative Disorders, Newcastle University, Newcastle, United Kingdom

**Keywords:** Treatment Resistant Depression, Difficult to treat depression, Depression

## Abstract

In this presentation a model for the management of difficult to treat depression (DTD) will be presented based upon a recently published international consensus statement (McAllister-Williams et al. 2020 Journal of Affective Disorders 267:264-282). This model emphasises the goals of: optimal symptom control – remission if possible; optimisation of psychosocial functioning; and optimisation of prophylaxis against relapse/deterioration in mood. Building on these goals, the model follows a number of principles. These include emphasizing the importance of shared decision making and measurement-based care, enhancing engagement and retention in services, self-management strategies and frequent re-assessments, all incorporated in an integrated service pathway. The model itself encompasses eight elements: 1. Optimal symptom control using conventional, guideline recommended, treatments but moving on to treatments beyond guidelines in an appropriate and timely way; 2. Targeting symptoms associated with poor outcomes, e.g. anxiety and pain; 3. Targeting symptoms associated with poor functioning and quality of life such as sleep difficulties, fatigue and cognitive dysfunction; 4. Screening for and managing physical, psychiatric, substance misuse and iatrogenic comorbidities; 5. Optimisation of long-term treatment; 6. Using self-management techniques to empower patients; 7. Using integrated health services to help provide a sense of containment and ensure wide consideration of treatment options; and 8. Establishing regular reviews of the patient’s diagnosis and treatment. Examples of each of these elements will be provided.

**Disclosure:**

In the last 5 years, R. Hamish McAllister-Williams has received fees from American Center for Psychiatry & Neurology United Arab Emirates, British Association for Psychopharmacology, European College of Neuropsychophamracology, International Society for A

